# Supplementary material for: Circulating Metabolites Associated with Prothrombin Time in Children with Congenital Heart Disease: An Untargeted Metabolomics Study
Source: Metabolites. 2026 Sep 1;16(9):640. doi: 10.3390/metabo16090640 (PMC13609389; doi:10.3390/metabo16090640)
Supplement: Supplementary file 1 [file metabolites-16-00640-s001.zip › metabolites-4410362-supplementary.pdf]

Circulating Metabolites Associated with Prothrombin Time and Activated Clotting Time in Children with Congenital Heart Disease: An Untargeted Metabolomics Study

Shengxu Li[1,2], Dave Watson[1,3], Alissa Jorgenson[4], Zainab Adelekan[1], Kathleen Garland[5], Benjamin Deonovic[1], Leah Zupancich[6], Weihong Tang[7], David M. Overman[2,6,8], Marnie T. Huntley[6,8]

1 Children's Minnesota Research Institute, Children's Minnesota, Minneapolis, MN 55404

2 Cardiovascular and Critical Care Research Center, Children's Minnesota, Minneapolis, MN 55404

3 Division of Clinical Trials and Biostatistics, Department of Quantitative Health Sciences, Mayo Clinic, Rochester, MN 55905

4 Clinical and Translational Science Institute, University of Minnesota, Minneapolis, MN 55414

5 Hematology and Oncology, Children's Minnesota, Minneapolis, MN 55404

6 The Children's Heart Clinic, Minneapolis, MN 55404

7 Division of Epidemiology and Community Health, School of Public Health, University of Minnesota, Minneapolis, MN 55454

8 Mayo Clinic-Children's Minnesota Cardiovascular Collaborative

Correspondence:

Shengxu Li, MD, PhD, MPH

Children's Minnesota Research Institute

2525 Chicago Avenue South

MS-40: LL08

Minneapolis, MN 55404

Email: Shengxu.li@childrensmn.org

And

Marnie T. Huntley, MD

The Children's Heart Clinic

2530 Chicago Avenue South, Suite 500

Minneapolis, MN 55404

Email: mhuntley@chc-pa.org

Figure S1

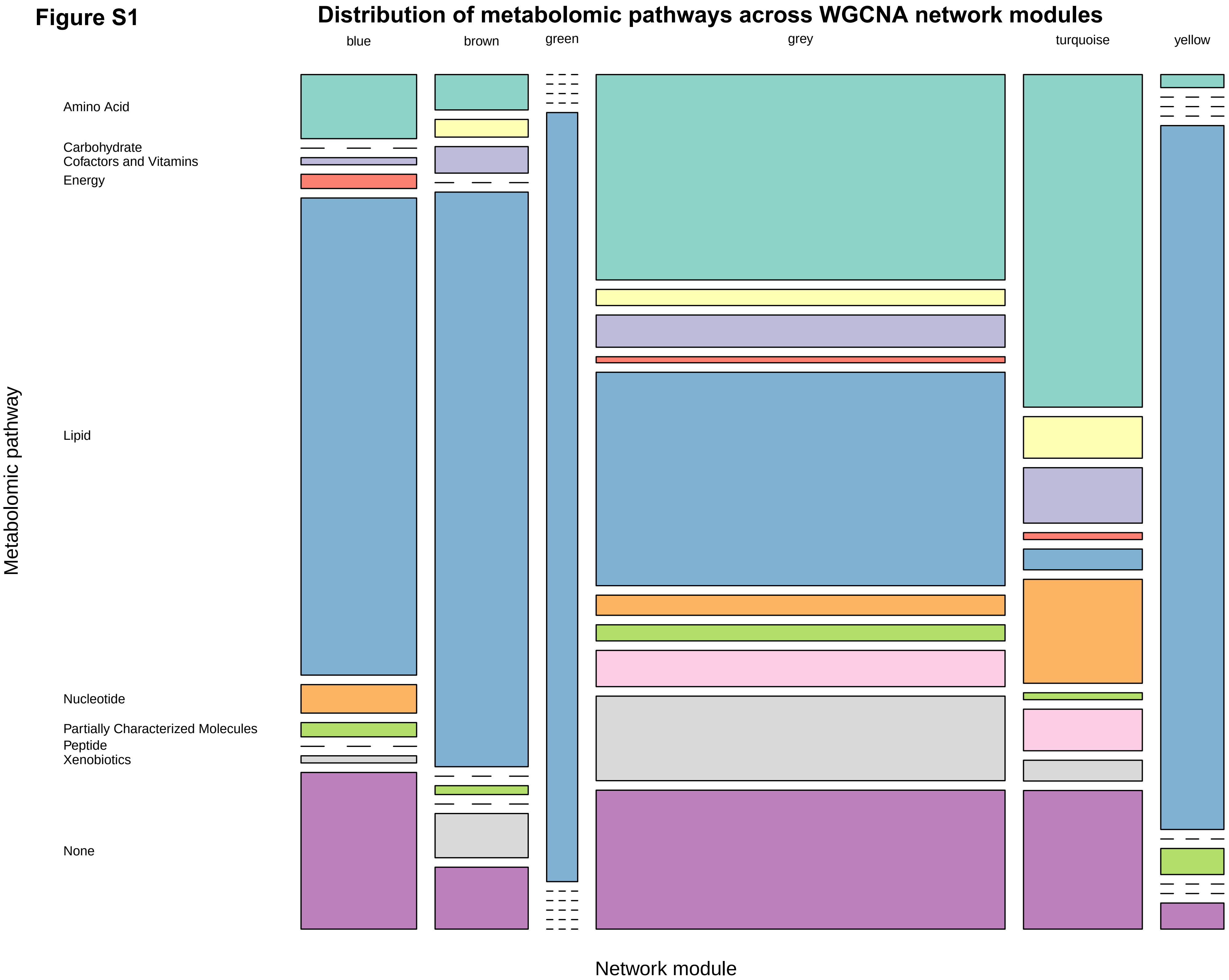
Distribution of metabolomic pathways across WGCNA network modules
blue
brown
green
grey
turquoise
yellow
Amino Acid
Carbohydrate
Cofactors and Vitamins
Energy
Lipid
Nucleotide
Partially Characterized Molecules
Peptide
Xenobiotics
None
Metabolomic pathway
Network module

Table S1. Membership of metabolites within each WGCNA module

Module Color	SUPER PATHWAY	SUB PATHWAY	CHEMICAL NAME	HMDB
blue	Lipid	Mevalonate Metabolism	3-hydroxy-3-methylglutarate	HMDB0000355
blue	Nucleotide	Purine Metabolism, Adenine containing	adenosine 3',5'-cyclic monophosphate (cAMP)	HMDB0000058
blue	Lipid	Ketone Bodies	3-hydroxybutyrate (BHBA)	HMDB0000442,HMDB0000357,HMDB0000011
blue	Nucleotide	Pyrimidine Metabolism, Orotate containing	orotate	HMDB0000226
blue	Amino Acid	Methionine, Cysteine, SAM and Taurine Metabolism	taurine	HMDB0000251
blue	Amino Acid	Glycine, Serine and Threonine Metabolism	betaine	HMDB0000043
blue	Amino Acid	Glycine, Serine and Threonine Metabolism	dimethylglycine	HMDB0000092
blue	Nucleotide	Purine Metabolism, (Hypo)Xanthine/Inosine containing	xanthine	HMDB0000292
blue	Nucleotide	Pyrimidine Metabolism, Thymine containing	3-aminoisobutyrate	HMDB0002166
blue	Energy	TCA Cycle	citrate	HMDB0000094
blue	Amino Acid	Tryptophan Metabolism	anthranilate	HMDB0001123
blue	Amino Acid	Leucine, Isoleucine and Valine Metabolism	methylsuccinate	HMDB0001844
blue	Amino Acid	Leucine, Isoleucine and Valine Metabolism	ethylmalonate	HMDB0000622
blue	Lipid	Fatty Acid, Dicarboxylate	suberate (C8-DC)	HMDB0000893
blue	Lipid	Fatty Acid, Monohydroxy	3-hydroxyoctanoate	HMDB0001954
blue	Lipid	Fatty Acid Metabolism (Acyl Carnitine, Long Chain Saturated)	palmitoylcarnitine (C16)	HMDB0000222
blue	Lipid	Fatty Acid Metabolism (Acyl Carnitine, Medium Chain)	hexanoylcarnitine (C6)	HMDB0000756
blue	Lipid	Fatty Acid Metabolism (Acyl Carnitine, Short Chain)	acetylcarnitine (C2)	HMDB0000201
blue	Lipid	Fatty Acid Metabolism (also BCAA Metabolism)	butyrylcarnitine (C4)	HMDB0002013
blue	Lipid	Fatty Acid, Dicarboxylate	dodecanedioate (C12-DC)	HMDB0000623
blue	Lipid	Fatty Acid, Monohydroxy	3-hydroxysebacate	HMDB0000350
blue	Lipid	Fatty Acid, Monohydroxy	5-hydroxyhexanoate	HMDB0000409,HMDB0000525
blue	Lipid	Fatty Acid, Dicarboxylate	sebacate (C10-DC)	HMDB0000792
blue	Lipid	Fatty Acid Metabolism (Acyl Carnitine, Medium Chain)	octanoylcarnitine (C8)	HMDB0000791
blue	Lipid	Fatty Acid Metabolism (Acyl Carnitine, Medium Chain)	decanoylcarnitine (C10)	HMDB0000651
blue	Lipid	Fatty Acid Metabolism (Acyl Carnitine, Long Chain Saturated)	myristoylcarnitine (C14)	HMDB0005066
blue	Energy	TCA Cycle	aconitate [cis or trans]	HMDB0000958,HMDB000072
blue	Lipid	Fatty Acid Metabolism (Acyl Carnitine, Long Chain Saturated)	stearoylcarnitine (C18)	HMDB0000848
blue	Lipid	Fatty Acid Metabolism (Acyl Carnitine, Medium Chain)	laurylcarnitine (C12)	HMDB000225
blue	Lipid	Fatty Acid, Dicarboxylate	3-hydroxydodecanedioate*	HMDB0000413
blue	Lipid	Fatty Acid Metabolism (Acyl Carnitine, Monounsaturated)	oleoylcarnitine (C18:1)	HMDB0005065
blue	Lipid	Fatty Acid, Dicarboxylate	tetradecanedioate (C14-DC)	HMDB0000872
blue	Lipid	Fatty Acid, Dicarboxylate	hexadecanedioate (C16-DC)	HMDB0000672
blue	Lipid	Fatty Acid, Dicarboxylate	octadecanedioate (C18-DC)	HMDB0000782
blue	Lipid	Carnitine Metabolism	deoxycarnitine	HMDB0001161
blue	Lipid	Fatty Acid Metabolism (Acyl Glutamine)	hexanoylglutamine	
blue	Cofactors and Vitamins	Hemoglobin and Porphyrin Metabolism	bilirubin (E,E)*	HMDB0240584
blue	Lipid	Secondary Bile Acid Metabolism	taurocholenate sulfate*	
blue	Xenobiotics	Food Component/Plant	cinnamoylglycine	HMDB0011621
blue	Lipid	Fatty Acid Metabolism (Acyl Carnitine, Monounsaturated)	cis-4-decenoylcarnitine (C10:1)	HMDB0013205
blue	Lipid	Fatty Acid, Dihydroxy	2S,3R-dihydroxybutyrate	HMDB0002453
blue	Lipid	Fatty Acid Metabolism (Acyl Carnitine, Polyunsaturated)	linoleoylcarnitine (C18:2)*	HMDB0006469
blue	Lipid	Fatty Acid Metabolism (Acyl Carnitine, Hydroxy)	(R)-3-hydroxybutyrylcarnitine	HMDB0013127
blue	Lipid	Fatty Acid Metabolism (Acyl Carnitine, Dicarboxylate)	octadecenedioylcarnitine (C18:1-DC)*	
blue	Lipid	Fatty Acid Metabolism (Acyl Carnitine, Dicarboxylate)	octadecanedioylcarnitine (C18-DC)*	
blue	Lipid	Fatty Acid Metabolism (Acyl Carnitine, Monounsaturated)	myristoleoylcarnitine (C14:1)*	HMDB0240588
blue	Lipid	Fatty Acid, Monohydroxy	3-hydroxyhexanoate	HMDB0061652,HMDB0010718
blue	Lipid	Fatty Acid Metabolism (Acyl Carnitine, Dicarboxylate)	adipoylcarnitine (C6-DC)	HMDB0061677
blue	Lipid	Fatty Acid Metabolism (Acyl Carnitine, Dicarboxylate)	suberoylcarnitine (C8-DC)	
blue	Amino Acid	Glutathione Metabolism	2-hydroxybutyrate/2-hydroxyisobutyrate	HMDB0000729,HMDB00008
blue	Lipid	Fatty Acid Metabolism (Acyl Carnitine, Hydroxy)	(S)-3-hydroxybutyrylcarnitine	HMDB0013127
blue	Lipid	Fatty Acid Metabolism (Acyl Carnitine, Monounsaturated)	palmitoleoylcarnitine (C16:1)*	HMDB0013207
blue	Lipid	Fatty Acid Metabolism (Acyl Carnitine, Dicarboxylate)	pimeloylcarnitine/3-methyladipoylcarnitine (C7-DC)	
blue	Lipid	Fatty Acid, Monohydroxy	2-hydroxynervonate*	
blue	Lipid	Endocannabinoid	N-oleoylserine	
blue	Lipid	Fatty Acid Metabolism (Acyl Carnitine, Polyunsaturated)	linolenoylcarnitine (C18:3)*	
blue	Lipid	Fatty Acid Metabolism (Acyl Carnitine, Long Chain Saturated)	behenoylcarnitine (C22)*	HMDB0062468
blue	Lipid	Fatty Acid Metabolism (Acyl Carnitine, Long Chain Saturated)	arachidoylcarnitine (C20)*	HMDB0006460
blue	Lipid	Fatty Acid Metabolism (Acyl Carnitine, Long Chain Saturated)	lignoceroylcarnitine (C24)*	HMDB0240665
blue	Lipid	Fatty Acid Metabolism (Acyl Carnitine, Long Chain Saturated)	cerotoylcarnitine (C26)*	HMDB0006347
blue	Lipid	Fatty Acid Metabolism (Acyl Carnitine, Monounsaturated)	ximenoylcarnitine (C26:1)*	
blue	Lipid	Fatty Acid Metabolism (Acyl Carnitine, Polyunsaturated)	arachidonoylcarnitine (C20:4)	HMDB0006455
blue	Lipid	Fatty Acid Metabolism (Acyl Carnitine, Monounsaturated)	eicosenoylcarnitine (C20:1)*	
blue	Lipid	Fatty Acid Metabolism (Acyl Carnitine, Polyunsaturated)	dihomo-linoleoylcarnitine (C20:2)*	
blue	Lipid	Fatty Acid Metabolism (Acyl Carnitine, Monounsaturated)	nervonoylcarnitine (C24:1)*	HMDB0006509
blue	Lipid	Fatty Acid Metabolism (Acyl Carnitine, Monounsaturated)	5-dodecenoylcarnitine (C12:1)	HMDB13326
blue	Lipid	Fatty Acid Metabolism (Acyl Carnitine, Hydroxy)	3-hydroxyoleoylcarnitine	
blue	Lipid	Fatty Acid Metabolism (Acyl Glycine)	trans-2-hexenoylglycine	
blue	Lipid	Fatty Acid, Dicarboxylate	dodecenedioate (C12:1-DC)*	HMDB0000933
blue	Lipid	Fatty Acid, Dicarboxylate	hexadecenedioate (C16:1-DC)*	
blue	Lipid	Fatty Acid, Dicarboxylate	octadecenedioate (C18:1-DC)	
blue	Lipid	Fatty Acid, Dicarboxylate	octadecadienedioate (C18:2-DC)*	
blue	Lipid	Fatty Acid Metabolism (Acyl Glycine)	3-hydroxybutyroylglycine**	
blue	Partially Characterized Molecules	Partially Characterized Molecules	glutamine conjugate of C6H10O2 (1)*	
blue	Partially Characterized Molecules	Partially Characterized Molecules	glutamine conjugate of C6H10O2 (2)*	
blue	Amino Acid	Tryptophan Metabolism	8-methoxykynurenate	HMDB0060426
blue	Lipid	Fatty Acid, Dicarboxylate	2-hydroxysebacate	HMDB0000424
blue	Lipid	Fatty Acid Metabolism (Acyl Carnitine, Hydroxy)	3-hydroxyhexanoylcarnitine (1)	
blue	Lipid	Fatty Acid, Dicarboxylate	tetradecadienedioate (C14:2-DC)*	
blue	Lipid	Fatty Acid Metabolism (Acyl Carnitine, Hydroxy)	3-hydroxydecanoylcarnitine	HMDB0061636
blue	Amino Acid	Methionine, Cysteine, SAM and Taurine Metabolism	succinoyltaurine	
blue	Lipid	Fatty Acid, Dicarboxylate	decadienedioic acid (C10:2-DC)**	
blue	Lipid	Fatty Acid, Branched	cis-3,4-methyleneheptanoate	
blue	Lipid	Fatty Acid Metabolism (Acyl Carnitine, Medium Chain)	cis-3,4-methyleneheptanoylcarnitine	
blue	Lipid	Fatty Acid Metabolism (Acyl Carnitine, Hydroxy)	3-hydroxyoctanoylcarnitine (1)	
blue	Lipid	Fatty Acid Metabolism (Acyl Carnitine, Hydroxy)	3-hydroxyoctanoylcarnitine (2)	
blue			X-11478	
blue			X-12101	
blue			X-12839	
blue			X-14939	
blue			X-15486	
blue			X-16397	
blue			X-16580	
blue			X-17335	
blue			X-17438	
blue			X-18886	
blue			X-18888	
blue			X-18913	
blue			X-18921	
blue			X-21364	
blue			X-21736	
blue			X-23665	
blue			X-23678	
blue			X-23680	
blue			X-24949	
blue			X-26106	
blue			X-26107	
blue			X-26108	
brown	Lipid	Sterol	cholesterol	HMDB0000067
brown	Lipid	Dihydroceramides	N-stearoyl-sphinganine (d18:0/18:0)*	HMDB0011761
brown	Lipid	Phosphatidylcholine (PC)	1-palmitoyl-2-linoleoyl-GPC (16:0/18:2)	HMDB0007973

brown	Lipid	Sphingomyelins	stearoyl sphingomyelin (d18:1/18:0)	HMDB0001348
brown	Lipid	Phosphatidylcholine (PC)	1-palmitoyl-2-oleoyl-GPC (16:0/18:1)	HMDB0007972
brown	Lipid	Ceramides	N-stearoyl-sphingosine (d18:1/18:0)*	HMDB0004950
brown	Carbohydrate	Glycolysis, Gluconeogenesis, and Pyruvate Metabolism	1,5-anhydroglucitol (1,5-AG)	HMDB0002712
brown	Lipid	Phosphatidylcholine (PC)	1,2-dipalmitoyl-GPC (16:0/16:0)	HMDB0000564
brown	Lipid	Phosphatidylcholine (PC)	1-myristoyl-2-palmitoyl-GPC (14:0/16:0)	HMDB0007869
brown	Carbohydrate	Fructose, Mannose and Galactose Metabolism	galactonate	HMDB0000565
brown	Lipid	Medium Chain Fatty Acid	10-undecenoate (11:1n1)	HMDB0033724
brown	Lipid	Lysophospholipid	1-palmitoyl-GPC (16:0)	HMDB0010382
brown	Xenobiotics	Food Component/Plant	homostachydrine*	HMDB0033433
brown	Amino Acid	Tryptophan Metabolism	tryptophan betaine	HMDB0061115
brown	Lipid	Hexosylceramides (HCER)	glycosyl-N-stearoyl-sphingosine (d18:1/18:0)	
brown	Cofactors and Vitamins	Vitamin A Metabolism	beta-cryptoxanthin	HMDB0033844
brown	Lipid	Sphingomyelins	sphingomyelin (d18:1/18:1, d18:2/18:0)	HMDB0012101
brown	Lipid	Sphingomyelins	palmitoyl sphingomyelin (d18:1/16:0)	HMDB0010169
brown	Xenobiotics	Food Component/Plant	ergothioneine	HMDB0003045
brown	Lipid	Fatty Acid, Dicarboxylate	tridecenedioate (C13:1-DC)*	
brown	Amino Acid	Methionine, Cysteine, SAM and Taurine Metabolism	S-methylcysteine	HMDB0002108
brown	Lipid	Lysoplasmalogen	1-(1-enyl-palmitoyl)-GPC (P-16:0)*	HMDB0010407
brown	Lipid	Lysoplasmalogen	1-(1-enyl-palmitoyl)-GPE (P-16:0)*	HMDB0011152
brown	Lipid	Lysoplasmalogen	1-(1-enyl-stearoyl)-GPE (P-18:0)*	HMDB0240598
brown	Lipid	Fatty Acid Metabolism (Acyl Carnitine, Long Chain Saturated)	margaroylcarnitine (C17)*	HMDB0006210
brown	Lipid	Sphingomyelins	sphingomyelin (d18:1/14:0, d16:1/16:0)*	HMDB0012097
brown	Lipid	Sphingomyelins	sphingomyelin (d18:2/16:0, d18:1/16:1)*	HMDB0240638,HMDB0240613
brown	Lipid	Sphingomyelins	sphingomyelin (d18:2/14:0, d18:1/14:1)*	HMDB0240637,HMDB0240612
brown	Lipid	Sphingomyelins	sphingomyelin (d18:1/24:1, d18:2/24:0)*	HMDB0012107
brown	Xenobiotics	Chemical	4-hydroxychlorothalonil	HMDB0240624
brown	Lipid	Sphingomyelins	sphingomyelin (d18:1/20:0, d16:1/22:0)*	HMDB0012102
brown	Lipid	Sphingomyelins	sphingomyelin (d18:1/20:1, d18:2/20:0)*	HMDB0240610,HMDB0240632
brown	Lipid	Sphingomyelins	behenoyl sphingomyelin (d18:1/22:0)*	HMDB0012103
brown	Lipid	Sphingomyelins	sphingomyelin (d18:1/22:2, d18:2/22:1, d16:1/24:2)*	HMDB0240670,HMDB0240672,HMDB0240669
brown	Lipid	Sphingomyelins	lignoceroyl sphingomyelin (d18:1/24:0)	HMDB0011697
brown	Lipid	Sphingomyelins	sphingomyelin (d17:1/16:0, d18:1/15:0, d16:1/17:0)*	HMDB0240617,HMDB0240608
brown	Lipid	Plasmalogen	1-(1-enyl-stearoyl)-2-oleoyl-GPE (P-18:0/18:1)	HMDB0011375
brown	Lipid	Sphingomyelins	sphingomyelin (d18:1/17:0, d17:1/18:0, d19:1/16:0)	HMDB0240620,HMDB0240609,HMDB0240622
brown	Lipid	Phosphatidylcholine (PC)	1-palmitoyl-2-stearoyl-GPC (16:0/18:0)	HMDB0007970
brown	Lipid	Dihydrosphingomyelins	palmitoyl dihydrosphingomyelin (d18:0/16:0)*	HMDB0010168
brown	Lipid	Sphingomyelins	tricosanoyl sphingomyelin (d18:1/23:0)*	HMDB0012105
brown	Lipid	Sphingomyelins	sphingomyelin (d18:2/23:0, d18:1/23:1, d17:1/24:1)*	HMDB0240634,HMDB0011696,HMDB0240614
brown	Lipid	Phosphatidylcholine (PC)	1-palmitoyl-2-palmitoleoyl-GPC (16:0/16:1)*	HMDB0007969
brown	Lipid	Plasmalogen	1-(1-enyl-stearoyl)-2-arachidonoyl-GPE (P-18:0/20:4)*	HMDB0005779
brown	Lipid	Plasmalogen	1-(1-enyl-palmitoyl)-2-arachidonoyl-GPE (P-16:0/20:4)*	HMDB0011352
brown	Lipid	Plasmalogen	1-(1-enyl-palmitoyl)-2-arachidonoyl-GPC (P-16:0/20:4)*	HMDB0011220
brown	Lipid	Sphingomyelins	sphingomyelin (d18:1/21:0, d17:1/22:0, d16:1/23:0)*	HMDB0240619,HMDB0240611,HMDB0240621
brown	Lipid	Dihydrosphingomyelins	behenoyl dihydrosphingomyelin (d18:0/22:0)*	HMDB0012091
brown	Lipid	Dihydrosphingomyelins	sphingomyelin (d18:0/18:0, d19:0/17:0)*	HMDB0012087
brown	Lipid	Dihydroceramides	N-palmitoyl-sphinganine (d18:0/16:0)	HMDB11760
brown	Lipid	Dihydrosphingomyelins	myristoyl dihydrosphingomyelin (d18:0/14:0)*	HMDB0012085
brown	Lipid	Plasmalogen	1-(1-enyl-palmitoyl)-2-palmitoyl-GPC (P-16:0/16:0)*	HMDB0011206
brown	Lipid	Plasmalogen	1-(1-enyl-stearoyl)-2-linoleoyl-GPE (P-18:0/18:2)*	HMDB0011376
brown	Lipid	Hexosylceramides (HCER)	glycosyl-N-palmitoyl-sphingosine (d18:1/16:0)	
brown	Lipid	Lactosylceramides (LCER)	lactosyl-N-behenoyl-sphingosine (d18:1/22:0)*	HMDB0011594
brown	Lipid	Hexosylceramides (HCER)	glycosyl-N-behenoyl-sphingadienine (d18:2/22:0)*	
brown	Lipid	Ceramides	ceramide (d16:1/24:1, d18:1/22:1)*	
brown	Lipid	Ceramides	N-palmitoyl-heptadecasphingosine (d17:1/16:0)*	HMDB0240685
brown	Lipid	Ceramides	ceramide (d18:2/24:1, d18:1/24:2)*	HMDB0240679,HMDB0240680
brown	Lipid	Hexosylceramides (HCER)	glycosyl ceramide (d18:2/24:1, d18:1/24:2)*	
brown	Lipid	Hexosylceramides (HCER)	glycosyl-N-tricosanoyl-sphingadienine (d18:2/23:0)*	
brown	Lipid	Hexosylceramides (HCER)	glycosyl ceramide (d18:1/23:1, d17:1/24:1)*	
brown	Lipid	Dihydrosphingomyelins	sphingomyelin (d18:0/20:0, d16:0/22:0)*	HMDB0012090
brown	Lipid	Sphingomyelins	sphingomyelin (d18:1/19:0, d19:1/18:0)*	
brown	Lipid	Sphingomyelins	sphingomyelin (d18:2/18:1)*	HMDB0001348
brown	Lipid	Sphingomyelins	sphingomyelin (d18:2/21:0, d16:2/23:0)*	HMDB0240676
brown	Lipid	Sphingomyelins	sphingomyelin (d18:2/23:1)*	HMDB0240668
brown	Lipid	Sphingomyelins	sphingomyelin (d18:1/25:0, d19:0/24:1, d20:1/23:0, d19:1/24:0)*	HMDB0240675,HMDB0240674,HMDB0240673,HMDB0240671
brown	Lipid	Sphingomyelins	sphingomyelin (d17:2/16:0, d18:2/15:0)*	HMDB0240677
brown	Lipid	Hexosylceramides (HCER)	glycosyl ceramide (d18:1/20:0, d16:1/22:0)*	
brown	Amino Acid	Lysine Metabolism	N,N,N-trimethyl-5-aminovalerate	
brown	Cofactors and Vitamins	Vitamin A Metabolism	carotene diol (1)	
brown	Cofactors and Vitamins	Vitamin A Metabolism	carotene diol (3)	
brown	Partially Characterized Molecules	Partially Characterized Molecules	glutamine conjugate of C7H12O2*	
brown	Lipid	Sphingomyelins	sphingomyelin (d17:1/14:0, d16:1/15:0)*	
brown	Amino Acid	Tryptophan Metabolism	6-bromotryptophan	
brown	Lipid	Fatty Acid Metabolism (Acyl Carnitine, Monounsaturated)	undecenoylcarnitine (C11:1)	
brown	Lipid	Ceramide PEs	palmitoyl-sphingosine-phosphoethanolamine (d18:1/16:0)	
brown	Xenobiotics	Chemical	3,5-dichloro-2,6-dihydroxybenzoic acid	
brown	Xenobiotics	Chemical	3-bromo-5-chloro-2,6-dihydroxybenzoic acid*	
brown			X-11381	
brown			X-11795	
brown			X-12798	
brown			X-18901	
brown			X-24522	
brown			X-24812	
brown			X-25420	
green	Lipid	Phosphatidylethanolamine (PE)	1-palmitoyl-2-oleoyl-GPE (16:0/18:1)	HMDB0005320
green	Lipid	Phosphatidylinositol (PI)	1-palmitoyl-2-linoleoyl-GPI (16:0/18:2)	HMDB0009784
green	Lipid	Phosphatidylinositol (PI)	1-stearoyl-2-arachidonoyl-GPI (18:0/20:4)	HMDB0009815
green	Lipid	Lysophospholipid	1-stearoyl-GPE (18:0)	HMDB0011130
green	Lipid	Lysophospholipid	1-arachidonoyl-GPC (20:4n6)*	HMDB0010395
green	Lipid	Lysophospholipid	1-palmitoyl-GPE (16:0)	HMDB0011503
green	Lipid	Lysophospholipid	1-arachidonoyl-GPE (20:4n6)*	HMDB0011517
green	Lipid	Phosphatidylethanolamine (PE)	1-stearoyl-2-oleoyl-GPE (18:0/18:1)	HMDB0008993
green	Lipid	Phosphatidylcholine (PC)	1-stearoyl-2-arachidonoyl-GPC (18:0/20:4)	HMDB0008048
green	Lipid	Phosphatidylethanolamine (PE)	1-palmitoyl-2-linoleoyl-GPE (16:0/18:2)	HMDB0005322
green	Lipid	Diacylglycerol	oleoyl-linoleoyl-glycerol (18:1/18:2) [1]	HMDB0007219
green	Lipid	Diacylglycerol	oleoyl-linoleoyl-glycerol (18:1/18:2) [2]	HMDB0007219
green	Lipid	Phosphatidylcholine (PC)	1-palmitoyl-2-arachidonoyl-GPC (16:0/20:4n6)	HMDB0007982
green	Lipid	Phosphatidylethanolamine (PE)	1-stearoyl-2-linoleoyl-GPE (18:0/18:2)*	HMDB0008994
green	Lipid	Phosphatidylethanolamine (PE)	1-stearoyl-2-arachidonoyl-GPE (18:0/20:4)	HMDB0009003
green	Lipid	Phosphatidylethanolamine (PE)	1-palmitoyl-2-arachidonoyl-GPE (16:0/20:4)*	HMDB0005323
green	Lipid	Phosphatidylethanolamine (PE)	1-palmitoyl-2-docosahexaenoyl-GPE (16:0/22:6)*	HMDB0008946
green	Lipid	Phosphatidylinositol (PI)	1-palmitoyl-2-arachidonoyl-GPI (16:0/20:4)*	HMDB0009789
green	Lipid	Diacylglycerol	palmitoyl-linoleoyl-glycerol (16:0/18:2) [2]*	HMDB0007103
green	Lipid	Diacylglycerol	oleoyl-oleoyl-glycerol (18:1/18:1) [1]*	HMDB0007218
green	Lipid	Diacylglycerol	oleoyl-oleoyl-glycerol (18:1/18:1) [2]*	HMDB0007218
green	Lipid	Diacylglycerol	linoleoyl-arachidonoyl-glycerol (18:2/20:4) [1]*	HMDB0007257
green	Lipid	Diacylglycerol	linoleoyl-arachidonoyl-glycerol (18:2/20:4) [2]*	HMDB0007257
green	Lipid	Diacylglycerol	linoleoyl-docosahexaenoyl-glycerol (18:2/22:6) [2]*	HMDB0007266
green	Lipid	Diacylglycerol	oleoyl-arachidonoyl-glycerol (18:1/20:4) [1]*	HMDB0007228
green	Lipid	Diacylglycerol	oleoyl-arachidonoyl-glycerol (18:1/20:4) [2]*	HMDB0007228
green	Lipid	Diacylglycerol	diacylglycerol (16:1/18:2 [2], 16:0/18:3 [1])*	
green	Lipid	Diacylglycerol	linoleoyl-linoleoyl-glycerol (18:2/18:2) [1]*	HMDB0007248

green	Lipid	Diacylglycerol	linoleoyl-linoleoyl-glycerol (18:2/18:2) [2]*	HMDB0007248
grey	Amino Acid	Polyamine Metabolism	spermidine	HMDB0001257
grey	Cofactors and Vitamins	Nicotinate and Nicotinamide Metabolism	1-methylnicotinamide	HMDB0000699
grey	Lipid	Fatty Acid, Dihydroxy	12,13-DiHOME	HMDB0004705
grey	Energy	TCA Cycle	alpha-ketoglutarate	HMDB0000208
grey	Amino Acid	Leucine, Isoleucine and Valine Metabolism	3-hydroxyisobutyrate	HMDB0000023,HMDB0000336
grey	Carbohydrate	Glycolysis, Gluconeogenesis, and Pyruvate Metabolism	3-phosphoglycerate	HMDB0000807
grey	Lipid	Primary Bile Acid Metabolism	cholate	HMDB0000619
grey	Nucleotide	Purine Metabolism, (Hypo)Xanthine/Inosine containing	hypoxanthine	HMDB0000157
grey	Lipid	Fatty Acid, Dihydroxy	9,10-DiHOME	HMDB0004704
grey	Nucleotide	Purine Metabolism, Adenine containing	adenosine 5'-monophosphate (AMP)	HMDB0000045
grey	Nucleotide	Purine Metabolism, Adenine containing	N6-methyladenosine	HMDB0004044
grey	Amino Acid	Urea cycle; Arginine and Proline Metabolism	arginine	HMDB0000517
grey	Amino Acid	Alanine and Aspartate Metabolism	aspartate	HMDB0000191
grey	Amino Acid	Tyrosine Metabolism	3-(4-hydroxyphenyl)lactate	HMDB0000755
grey	Amino Acid	Phenylalanine Metabolism	phenylpyruvate	HMDB0000205
grey	Amino Acid	Histidine Metabolism	carnosine	HMDB0000033
grey	Cofactors and Vitamins	Hemoglobin and Porphyrin Metabolism	biliverdin	HMDB0001008
grey	Lipid	Phospholipid Metabolism	choline phosphate	HMDB0001565
grey	Lipid	Corticosteroids	cortisone	HMDB0002802
grey	Amino Acid	Glutathione Metabolism	cysteinylglycine	HMDB0000078
grey	Lipid	Sphingosines	sphingosine	HMDB0000252
grey	Peptide	Gamma-glutamyl Amino Acid	gamma-glutamylglutamate	HMDB0011737
grey	Xenobiotics	Food Component/Plant	gluconate	HMDB0000625
grey	Amino Acid	Glycine, Serine and Threonine Metabolism	glycine	HMDB0000123
grey	Lipid	Primary Bile Acid Metabolism	glycocholate	HMDB0000138
grey	Amino Acid	Creatine Metabolism	guanidinoacetate	HMDB0000128
grey	Amino Acid	Histidine Metabolism	histidine	HMDB0000177
grey	Lipid	Corticosteroids	cortisol	HMDB0000063
grey	Amino Acid	Methionine, Cysteine, SAM and Taurine Metabolism	hypotaurine	HMDB0000965
grey	Nucleotide	Purine Metabolism, (Hypo)Xanthine/Inosine containing	inosine	HMDB0000195
grey	Lipid	Inositol Metabolism	myo-inositol	HMDB0000211
grey	Amino Acid	Leucine, Isoleucine and Valine Metabolism	isoleucine	HMDB0000172
grey	Amino Acid	Lysine Metabolism	2-aminoadipate	HMDB0000510
grey	Amino Acid	Urea cycle; Arginine and Proline Metabolism	citrulline	HMDB0000904
grey	Amino Acid	Leucine, Isoleucine and Valine Metabolism	leucine	HMDB0000687
grey	Amino Acid	Lysine Metabolism	lysine	HMDB0003405
grey	Energy	TCA Cycle	malate	HMDB0031518,HMDB0000156,HMDB0000744
grey	Amino Acid	Methionine, Cysteine, SAM and Taurine Metabolism	methionine	HMDB0000696
grey	Lipid	Fatty Acid Metabolism (also BCAA Metabolism)	methylmalonate (MMA)	HMDB0000202
grey	Cofactors and Vitamins	Nicotinate and Nicotinamide Metabolism	nicotinamide	HMDB0001406
grey	Lipid	Medium Chain Fatty Acid	pelargonate (9:0)	HMDB0000847
grey	Amino Acid	Urea cycle; Arginine and Proline Metabolism	ornithine	HMDB0000214
grey	Amino Acid	Phenylalanine Metabolism	phenylalanine	HMDB0000159
grey	Energy	Oxidative Phosphorylation	phosphate	HMDB0001429
grey	Xenobiotics	Food Component/Plant	phytanate	HMDB0000801
grey	Amino Acid	Urea cycle; Arginine and Proline Metabolism	proline	HMDB0000162,HMDB0003411
grey	Carbohydrate	Glycolysis, Gluconeogenesis, and Pyruvate Metabolism	lactate	HMDB0000190
grey	Cofactors and Vitamins	Vitamin B6 Metabolism	pyridoxal	HMDB0001545
grey	Xenobiotics	Drug - Topical Agents	salicylate	HMDB0001895
grey	Amino Acid	Glycine, Serine and Threonine Metabolism	serine	HMDB0000187
grey	Nucleotide	Pyrimidine Metabolism, Uracil containing	uridine	HMDB0000296
grey	Nucleotide	Pyrimidine Metabolism, Uracil containing	2'-deoxyuridine	HMDB0000012
grey	Amino Acid	Histidine Metabolism	trans-urocanate	HMDB0000301
grey	Amino Acid	Glutamate Metabolism	glutamate	HMDB0000148
grey	Amino Acid	Glutamate Metabolism	glutamine	HMDB0000641
grey	Amino Acid	Glycine, Serine and Threonine Metabolism	threonine	HMDB0000167
grey	Amino Acid	Tryptophan Metabolism	tryptophan	HMDB0000929
grey	Amino Acid	Leucine, Isoleucine and Valine Metabolism	valine	HMDB0000883
grey	Carbohydrate	Glycolysis, Gluconeogenesis, and Pyruvate Metabolism	glucose	HMDB0000122
grey	Carbohydrate	Fructose, Mannose and Galactose Metabolism	mannose	HMDB0000169
grey	Amino Acid	Alanine and Aspartate Metabolism	alanine	HMDB0000161
grey	Amino Acid	Tyrosine Metabolism	tyrosine	HMDB0000158
grey	Carbohydrate	Glycolysis, Gluconeogenesis, and Pyruvate Metabolism	pyruvate	HMDB0000243
grey	Nucleotide	Pyrimidine Metabolism, Thymine containing	thymidine	HMDB0000273
grey	Amino Acid	Alanine and Aspartate Metabolism	asparagine	HMDB0000168
grey	Lipid	Medium Chain Fatty Acid	caprylate (8:0)	HMDB0000482
grey	Amino Acid	Urea cycle; Arginine and Proline Metabolism	trans-4-hydroxyproline	HMDB0000725
grey	Amino Acid	Glutathione Metabolism	5-oxoproline	HMDB0000267
grey	Amino Acid	Tryptophan Metabolism	picolinate	HMDB0002243
grey	Amino Acid	Glycine, Serine and Threonine Metabolism	sarcosine	HMDB0000271
grey	Amino Acid	Lysine Metabolism	pipecolate	HMDB0000070
grey	Lipid	Phospholipid Metabolism	phosphoethanolamine	HMDB0000224
grey	Cofactors and Vitamins	Hemoglobin and Porphyrin Metabolism	bilirubin (Z,Z)	HMDB0000054
grey	Amino Acid	Tyrosine Metabolism	thyroxine	HMDB0000248
grey	Peptide	Gamma-glutamyl Amino Acid	gamma-glutamyltyrosine	HMDB0011741
grey	Cofactors and Vitamins	Tocopherol Metabolism	alpha-tocopherol	HMDB0001893
grey	Amino Acid	Glutathione Metabolism	2-aminobutyrate	HMDB0000452
grey	Peptide	Gamma-glutamyl Amino Acid	gamma-glutamylglutamine	HMDB0011738
grey	Amino Acid	Tyrosine Metabolism	4-hydroxyphenylpyruvate	HMDB0000707
grey	Lipid	Short Chain Fatty Acid	butyrate/isobutyrate (4:0)	HMDB0000039
grey	Amino Acid	Creatine Metabolism	creatine	HMDB0000064
grey	Amino Acid	Tyrosine Metabolism	3-methoxytyrosine	HMDB0001434
grey	Lipid	Primary Bile Acid Metabolism	glycochenodeoxycholate	HMDB0000637
grey	Lipid	Primary Bile Acid Metabolism	taurochenodeoxycholate	HMDB0000951
grey	Lipid	Primary Bile Acid Metabolism	taurocholate	HMDB0000036
grey	Xenobiotics	Benzoate Metabolism	2-hydroxyhippurate (salicylurate)	HMDB0000840
grey	Lipid	Carnitine Metabolism	carnitine	HMDB0000062
grey	Xenobiotics	Benzoate Metabolism	hippurate	HMDB0000714
grey	Amino Acid	Tryptophan Metabolism	xanthurenate	HMDB0000881
grey	Amino Acid	Leucine, Isoleucine and Valine Metabolism	3-methyl-2-oxovalerate	HMDB0000491
grey	Amino Acid	Methionine, Cysteine, SAM and Taurine Metabolism	methionine sulfoxide	HMDB0002005
grey	Amino Acid	Histidine Metabolism	3-methylhistidine	HMDB0000479
grey	Amino Acid	Lysine Metabolism	5-hydroxylysine	HMDB0000450
grey	Lipid	Glycerolipid Metabolism	glycerol 3-phosphate	HMDB0000126
grey	Lipid	Phospholipid Metabolism	glycerophosphorylcholine (GPC)	HMDB0000086
grey	Amino Acid	Glutamate Metabolism	N-acetylglutamate	HMDB0001138
grey	Carbohydrate	Pentose Metabolism	ribitol	HMDB0002917,HMDB0001851,HMDB0000568,HMDB0000508
grey	Amino Acid	Tryptophan Metabolism	indolelactate	HMDB0000671
grey	Amino Acid	Tryptophan Metabolism	3-indoxyl sulfate	HMDB0000682
grey	Amino Acid	Leucine, Isoleucine and Valine Metabolism	4-methyl-2-oxopentanoate	HMDB0000695
grey	Lipid	Sphingosines	sphingosine 1-phosphate	HMDB0000277
grey	Lipid	Phosphatidylserine (PS)	1-stearoyl-2-oleoyl-GPS (18:0/18:1)	HMDB0010163
grey	Lipid	Lysophospholipid	1-stearoyl-GPI (18:0)	HMDB0240261
grey	Xenobiotics	Benzoate Metabolism	4-acetylphenol sulfate	
grey	Lipid	Fatty Acid, Monohydroxy	2-hydroxyoctanoate	HMDB0002264
grey	Amino Acid	Phenylalanine Metabolism	phenyllactate (PLA)	HMDB0000779,HMDB0000563
grey	Lipid	Androgenic Steroids	dehydroepiandrosterone sulfate (DHEA-S)	HMDB0001032
grey	Amino Acid	Methionine, Cysteine, SAM and Taurine Metabolism	cysteine s-sulfate	HMDB0000731
grey	Xenobiotics	Food Component/Plant	tartronate (hydroxymalonate)	HMDB0035227
grey	Cofactors and Vitamins	Ascorbate and Aldarate Metabolism	oxalate (ethanedioate)	HMDB0002329
grey	Xenobiotics	Chemical	iminodiacetate (IDA)	HMDB0011753

grey	Amino Acid	Leucine, Isoleucine and Valine Metabolism	3-methyl-2-oxobutyrate	HMDB0000019
grey	Amino Acid	Urea cycle; Arginine and Proline Metabolism	homoarginine	HMDB0000670
grey	Lipid	Monoacylglycerol	2-linoleoylglycerol (18:2)	HMDB0011538
grey	Xenobiotics	Chemical	EDTA	HMDB0015109
grey	Cofactors and Vitamins	Ascorbate and Aldarate Metabolism	threonate	HMDB0062620,HMDB0000943
grey	Amino Acid	Tryptophan Metabolism	indoleacetate	HMDB0000197
grey	Amino Acid	Leucine, Isoleucine and Valine Metabolism	isobutyrylcarnitine (C4)	HMDB0000736
grey	Cofactors and Vitamins	Nicotinate and Nicotinamide Metabolism	trigonelline (N'-methylnicotinate)	HMDB0000875
grey	Amino Acid	Tyrosine Metabolism	N-acetyltyrosine	HMDB0000866
grey	Lipid	Fatty Acid Metabolism (also BCAA Metabolism)	propionylcarnitine (C3)	HMDB0000824
grey	Amino Acid	Urea cycle; Arginine and Proline Metabolism	pro-hydroxy-pro	HMDB0006695
grey	Lipid	Fatty Acid, Dicarboxylate	3-carboxy-4-methyl-5-propyl-2-furanpropanoate (CMPF)	HMDB0061112
grey	Amino Acid	Glutamate Metabolism	N-acetylglutamine	HMDB0006029
grey	Amino Acid	Tryptophan Metabolism	N-acetyltryptophan	HMDB0013713
grey	Amino Acid	Alanine and Aspartate Metabolism	N-acetylasparagine	HMDB0006028
grey	Amino Acid	Urea cycle; Arginine and Proline Metabolism	N-acetylarginine	HMDB0004620
grey	Lipid	Lysophospholipid	1-stearoyl-GPC (18:0)	HMDB0010384
grey	Lipid	Lysophospholipid	1-oleoyl-GPC (18:1)	HMDB0002815
grey	Lipid	Secondary Bile Acid Metabolism	hyocholate	HMDB0000760
grey	Amino Acid	Histidine Metabolism	N-acetylhistidine	HMDB0032055
grey	Peptide	Gamma-glutamyl Amino Acid	gamma-glutamylglycine	HMDB0011667
grey	Xenobiotics	Food Component/Plant	stachydrine	HMDB0004827
grey	Amino Acid	Leucine, Isoleucine and Valine Metabolism	alpha-hydroxyisovalerate	HMDB0000407
grey	Peptide	Gamma-glutamyl Amino Acid	gamma-glutamylmethionine	HMDB0029155
grey	Peptide	Gamma-glutamyl Amino Acid	gamma-glutamylthreonine	HMDB0029159
grey	Xenobiotics	Benzoate Metabolism	p-cresol sulfate	HMDB0011635
grey	Cofactors and Vitamins	Hemoglobin and Porphyrin Metabolism	heme	HMDB0003178
grey	Amino Acid	Leucine, Isoleucine and Valine Metabolism	isovalerylcarnitine (C5)	HMDB0000688
grey	Lipid	Lysophospholipid	1-linoleoyl-GPC (18:2)	HMDB0010386
grey	Nucleotide	Pyrimidine Metabolism, Cytidine containing	N4-acetylcytidine	HMDB0005923
grey	Peptide	Acetylated Peptides	phenylacetylglutamine	HMDB0006344
grey	Xenobiotics	Benzoate Metabolism	4-hydroxyhippurate	HMDB0013678
grey	Amino Acid	Glutathione Metabolism	cysteine-glutathione disulfide	HMDB0000656
grey	Nucleotide	Pyrimidine Metabolism, Uracil containing	5-methyluridine (ribothymidine)	HMDB0000884
grey	Nucleotide	Pyrimidine Metabolism, Cytidine containing	3-methylcytidine	HMDB0240577
grey	Amino Acid	Tyrosine Metabolism	phenol sulfate	HMDB0060015
grey	Lipid	Lysophospholipid	1-palmitoleoyl-GPC (16:1)*	HMDB0010383
grey	Amino Acid	Leucine, Isoleucine and Valine Metabolism	2-hydroxy-3-methylvalerate	HMDB0000317
grey	Lipid	Lysophospholipid	2-palmitoyl-GPC (16:0)*	HMDB0061702
grey	Lipid	Lysophospholipid	1-oleoyl-GPE (18:1)	HMDB0011506
grey	Lipid	Lysophospholipid	1-linoleoyl-GPE (18:2)*	HMDB0011507
grey	Amino Acid	Leucine, Isoleucine and Valine Metabolism	beta-hydroxyisovaleroylcarnitine	
grey	Xenobiotics	Drug - Topical Agents	hydroquinone sulfate	HMDB0240263
grey	Xenobiotics	Benzoate Metabolism	catechol sulfate	HMDB0059724
grey	Lipid	Sterol	cholesterol sulfate	HMDB00653
grey	Amino Acid	Glutamate Metabolism	N-acetyl-aspartyl-glutamate (NAAG)	HMDB0001067
grey	Lipid	Phospholipid Metabolism	glycerophosphoethanolamine	HMDB0000114
grey	Lipid	Lysophospholipid	1-palmitoyl-GPI (16:0)	HMDB0061695
grey	Lipid	Secondary Bile Acid Metabolism	glycolithocholate sulfate*	HMDB0002639
grey	Lipid	Secondary Bile Acid Metabolism	taurolithocholate 3-sulfate	HMDB0002580
grey	Carbohydrate	Fructose, Mannose and Galactose Metabolism	mannitol/sorbitol	HMDB0000247,HMDB0000765
grey	Xenobiotics	Benzoate Metabolism	4-vinylphenol sulfate	HMDB0062775
grey	Xenobiotics	Benzoate Metabolism	4-ethylphenylsulfate	HMDB0062551
grey	Xenobiotics	Food Component/Plant	thymol sulfate	HMDB0062720
grey	Lipid	Lysophospholipid	1-oleoyl-GPI (18:1)	HMDB0061693
grey	Xenobiotics	Benzoate Metabolism	o-cresol sulfate	HMDB0011635
grey	Xenobiotics	Food Component/Plant	4-allylphenol sulfate	
grey	Lipid	Sphingolipid Synthesis	sphinganine-1-phosphate	HMDB0001383
grey	Amino Acid	Urea cycle; Arginine and Proline Metabolism	N-methylproline	HMDB0094696
grey	Lipid	Progestin Steroids	5alpha-pregnan-3beta,20alpha-diol disulfate	HMDB0094650
grey	Lipid	Secondary Bile Acid Metabolism	glycocholenate sulfate*	
grey	Lipid	Androgenic Steroids	androstenediol (3beta,17beta) disulfate (1)	HMDB0240313
grey	Lipid	Pregnenolone Steroids	pregnenediol disulfate (C21H34O8S2)*	
grey	Lipid	Androgenic Steroids	androstenediol (3beta,17beta) disulfate (2)	HMDB0240313
grey	Lipid	Pregnenolone Steroids	21-hydroxypregnenolone disulfate	
grey	Lipid	Pregnenolone Steroids	pregnenediol sulfate (C21H34O5S)*	HMDB0000774
grey	Cofactors and Vitamins	Tocopherol Metabolism	gamma-CEHC	HMDB0001931
grey	Lipid	Androgenic Steroids	16alpha-hydroxy DHEA 3-sulfate	HMDB0062544
grey	Lipid	Pregnenolone Steroids	pregnenolone sulfate	HMDB0000774
grey	Lipid	Androgenic Steroids	andro steroid monosulfate C19H28O6S (1)*	HMDB0002759
grey	Amino Acid	Tryptophan Metabolism	indole-3-carboxylate	HMDB0003320
grey	Xenobiotics	Food Component/Plant	2,3-dihydroxyisovalerate	HMDB0012141
grey	Lipid	Secondary Bile Acid Metabolism	isoursodeoxycholate	HMDB0000686
grey	Amino Acid	Histidine Metabolism	formiminoglutamate	HMDB0000854
grey	Amino Acid	Glutamate Metabolism	4-hydroxyglutamate	HMDB0001344
grey	Cofactors and Vitamins	Pantothenate and CoA Metabolism	pantoate	HMDB0240389
grey	Amino Acid	Urea cycle; Arginine and Proline Metabolism	argininate*	HMDB0003148
grey	Amino Acid	Urea cycle; Arginine and Proline Metabolism	2-oxoarginine*	HMDB0004225
grey	Lipid	Lysophospholipid	1-lignoceroyl-GPC (24:0)	HMDB0010405
grey	Amino Acid	Histidine Metabolism	1-methyl-5-imidazoleacetate	HMDB04988
grey	Lipid	Secondary Bile Acid Metabolism	glycoursodeoxycholate	HMDB0000708
grey	Amino Acid	Methionine, Cysteine, SAM and Taurine Metabolism	S-methylcysteine sulfoxide	HMDB0029432
grey	Lipid	Fatty Acid, Dicarboxylate	eicosanedioate (C20-DC)	
grey	Lipid	Fatty Acid, Dicarboxylate	docosadioate (C22-DC)	
grey	Peptide	Dipeptide	isoleucylglycine	HMDB0028907
grey	Peptide	Dipeptide	valylleucine	HMDB0029131
grey	Amino Acid	Glutamate Metabolism	beta-citrylglutamate	
grey	Lipid	Phospholipid Metabolism	trimethylamine N-oxide	HMDB0000925
grey	Amino Acid	Lysine Metabolism	N6-methyllysine	HMDB0002038
grey	Amino Acid	Histidine Metabolism	imidazole propionate	HMDB0002271
grey	Peptide	Dipeptide	phenylalanylglycine	HMDB0028995
grey	Peptide	Dipeptide	valylglutamine	HMDB0029125
grey	Peptide	Dipeptide	valylglycine	HMDB0029127
grey	Lipid	Lysophospholipid	2-stearoyl-GPE (18:0)*	HMDB0011129
grey	Lipid	Secondary Bile Acid Metabolism	glycohyocholate	HMDB0000138
grey	Lipid	Fatty Acid, Monohydroxy	2-hydroxydecanoate	HMDB0094656
grey	Xenobiotics	Benzoate Metabolism	4-methylcatechol sulfate	HMDB0240459
grey	Xenobiotics	Benzoate Metabolism	3-methyl catechol sulfate (1)	
grey	Xenobiotics	Benzoate Metabolism	guaiacol sulfate	HMDB0060013
grey	Cofactors and Vitamins	Tocopherol Metabolism	gamma-CEHC glucuronide*	
grey	Xenobiotics	Food Component/Plant	2-piperidinone	HMDB0011749
grey	Xenobiotics	Food Component/Plant	2-aminophenol sulfate	HMDB0061116
grey	Amino Acid	Urea cycle; Arginine and Proline Metabolism	N-delta-acetylornithine	HMDB0003357
grey	Amino Acid	Polyamine Metabolism	acisoga	HMDB0061384
grey	Lipid	Fatty Acid, Amino	2-aminoheptanoate	HMDB0094649
grey	Amino Acid	Tyrosine Metabolism	3-methoxytyramine sulfate	
grey	Lipid	Lysophospholipid	1-linolenoyl-GPC (18:3)*	HMDB0010388
grey	Amino Acid	Lysine Metabolism	fructosyllysine	HMDB0034879
grey	Amino Acid	Leucine, Isoleucine and Valine Metabolism	3-methylglutarylcarnitine (2)	HMDB0000552
grey	Xenobiotics	Food Component/Plant	2-keto-3-deoxy-gluconate	HMDB0001353
grey	Amino Acid	Tyrosine Metabolism	N-formylphenylalanine	HMDB0240317
grey	Xenobiotics	Chemical	3-hydroxypyridine sulfate	

grey	Peptide	Acetylated Peptides	phenylacetylcarnitine	
grey	Xenobiotics	Benzoate Metabolism	methyl-4-hydroxybenzoate sulfate	HMDB0041646
grey	Xenobiotics	Benzoate Metabolism	propyl 4-hydroxybenzoate	HMDB0032574
grey	Xenobiotics	Chemical	6-hydroxyindole sulfate	HMDB0000682
grey	Amino Acid	Tyrosine Metabolism	4-methoxyphenol sulfate	
grey	Xenobiotics	Benzoate Metabolism	propyl 4-hydroxybenzoate sulfate	HMDB0135261
grey	Xenobiotics	Food Component/Plant	umbelliferone sulfate	HMDB0240565
grey	Lipid	Sphingomyelins	sphingomyelin (d18:1/20:2, d18:2/20:1, d16:1/22:2)*	
grey	Amino Acid	Tyrosine Metabolism	dopamine 3-O-sulfate	HMDB0006275
grey	Xenobiotics	Benzoate Metabolism	3-methoxycatechol sulfate (2)	
grey	Amino Acid	Tryptophan Metabolism	N-acetylkynurenine (2)	HMDB0240342
grey	Lipid	Primary Bile Acid Metabolism	glycochenodeoxycholate 3-sulfate	HMDB0002409,HMDB0002496,HMDB0002497
grey	Xenobiotics	Drug - Neurological	rocuronium	HMDB0014866
grey	Lipid	Phosphatidylcholine (PC)	1,2-dilinoleoyl-GPC (18:2/18:2)	HMDB0008138
grey	Lipid	Phosphatidylcholine (PC)	1-stearoyl-2-oleoyl-GPC (18:0/18:1)	HMDB0008038
grey	Lipid	Phosphatidylcholine (PC)	1-palmitoyl-2-docosahexaenoyl-GPC (16:0/22:6)	HMDB0007991
grey	Lipid	Phosphatidylcholine (PC)	1-stearoyl-2-docosahexaenoyl-GPC (18:0/22:6)	HMDB0008057
grey	Lipid	Monoacylglycerol	1-palmitoleoylglycerol (16:1)*	HMDB0011565
grey	Lipid	Sphingomyelins	sphingomyelin (d18:2/24:1, d18:1/24:2)*	HMDB0240636,HMDB0240615
grey	Lipid	Phosphatidylcholine (PC)	1-stearoyl-2-linoleoyl-GPC (18:0/18:2)*	HMDB0008039
grey	Lipid	Phosphatidylinositol (PI)	1-stearoyl-2-linoleoyl-GPI (18:0/18:2)	HMDB0009809
grey	Cofactors and Vitamins	Tocopherol Metabolism	gamma-tocopherol/beta-tocopherol	HMDB0006335,HMDB0001492
grey	Lipid	Plasmalogen	1-(1-enyl-palmitoyl)-2-oleoyl-GPE (P-16:0/18:1)*	HMDB0011342
grey	Lipid	Plasmalogen	1-(1-enyl-palmitoyl)-2-oleoyl-GPC (P-16:0/18:1)*	HMDB0007996
grey	Lipid	Plasmalogen	1-(1-enyl-palmitoyl)-2-linoleoyl-GPC (P-16:0/18:2)*	HMDB0011211
grey	Lipid	Lactosylceramides (LCER)	lactosyl-N-palmitoyl-sphingosine (d18:1/16:0)	HMDB0006750
grey	Amino Acid	Tryptophan Metabolism	5-hydroxyindole sulfate	
grey	Lipid	Phosphatidylinositol (PI)	1-palmitoyl-2-oleoyl-GPI (16:0/18:1)*	HMDB0009783
grey	Lipid	Plasmalogen	1-(1-enyl-palmitoyl)-2-linoleoyl-GPE (P-16:0/18:2)*	HMDB0011343
grey	Lipid	Lysophospholipid	1-linoleoyl-GPA (18:2)*	HMDB0007856
grey	Lipid	Phosphatidylcholine (PC)	1-oleoyl-2-docosahexaenoyl-GPC (18:1/22:6)*	HMDB0008123
grey	Lipid	Phosphatidylcholine (PC)	1-linoleoyl-2-arachidonoyl-GPC (18:2/20:4n6)*	HMDB0008147
grey	Lipid	Phosphatidylcholine (PC)	1-myristoyl-2-arachidonoyl-GPC (14:0/20:4)*	HMDB0007883
grey	Lipid	Plasmalogen	1-(1-enyl-palmitoyl)-2-palmitoleoyl-GPC (P-16:0/16:1)*	HMDB0011207
grey	Lipid	Phosphatidylinositol (PI)	1-stearoyl-2-oleoyl-GPI (18:0/18:1)*	HMDB0240667
grey	Lipid	Lysophospholipid	1-linoleoyl-GPG (18:2)*	HMDB0240600
grey	Lipid	Fatty Acid Metabolism (Acyl Choline)	palmitoylcholine	HMDB0240592
grey	Cofactors and Vitamins	Ascorbate and Aldarate Metabolism	ascorbic acid 2-sulfate	HMDB0060649
grey	Lipid	Fatty Acid Metabolism (Acyl Choline)	arachidonoylcholine	HMDB0240583
grey	Lipid	Phosphatidylcholine (PC)	1-linoleoyl-2-linolenoyl-GPC (18:2/18:3)*	HMDB0008141
grey	Lipid	Phosphatidylcholine (PC)	1-palmitoleoyl-2-linolenoyl-GPC (16:1/18:3)*	HMDB0008008
grey	Peptide	Gamma-glutamyl Amino Acid	gamma-glutamyl-alpha-lysine	
grey	Lipid	Lactosylceramides (LCER)	lactosyl-N-nervonoyl-sphingosine (d18:1/24:1)*	HMDB0004872
grey	Lipid	Hexosylceramides (HCER)	glycosyl-N-(2-hydroxynervonoyl)-sphingosine (d18:1/24:1(2OH))*	
grey	Lipid	Fatty Acid Metabolism (Acyl Choline)	linoleoylcholine*	HMDB0013213
grey	Lipid	Sphingomyelins	sphingomyelin (d18:2/24:2)*	HMDB0240644
grey	Amino Acid	Polyamine Metabolism	(N(1) + N(8))-acetylspermidine	HMDB0002189,HMDB0001276
grey	Lipid	Fatty Acid, Dicarboxylate	hydroxy-CMPF*	
grey	Carbohydrate	Pentose Metabolism	lyxonate	HMDB0060255
grey	Lipid	Fatty Acid, Dicarboxylate	3-carboxy-4-methyl-5-pentyl-2-furanpropionate (3-CMPFP)**	HMDB0061643
grey	Lipid	Fatty Acid, Amino	N-acetyl-2-aminooctanoate*	HMDB0059745
grey	Xenobiotics	Chemical	perfluorooctanoate (PFOA)	HMDB0059587
grey	Xenobiotics	Food Component/Plant	3-formylindole	HMDB29737
grey	Peptide	Gamma-glutamyl Amino Acid	gamma-glutamylcitrulline*	
grey	Partially Characterized Molecules	Partially Characterized Molecules	glycine conjugate of C10H14O2 (1)*	
grey	Xenobiotics	Food Component/Plant	ethyl beta-glucopyranoside	HMDB0029968
grey	Cofactors and Vitamins	Tocopherol Metabolism	delta-CEHC	
grey	Amino Acid	Lysine Metabolism	N6,N6-dimethyllysine	HMDB0013287
grey	Amino Acid	Tryptophan Metabolism	indoleacetoylcarnitine*	
grey	Xenobiotics	Chemical	2-naphthol sulfate	
grey	Amino Acid	Lysine Metabolism	N2-acetyl,N6,N6-dimethyllysine	
grey	Amino Acid	Lysine Metabolism	N2-acetyl,N6-methyllysine	
grey	Lipid	Secondary Bile Acid Metabolism	deoxycholic acid glucuronide	
grey	Xenobiotics	Food Component/Plant	methyl vanillate sulfate	
grey	Lipid	Secondary Bile Acid Metabolism	glycoursodeoxycholic acid sulfate (1)	
grey	Amino Acid	Leucine, Isoleucine and Valine Metabolism	2-ketocaprylate	HMDB13211
grey	Xenobiotics	Drug - Topical Agents	2,6-dihydroxybenzoic acid	HMDB0013676
grey	Lipid	Sphingomyelins	hydroxypalmitoyl sphingomyelin (d18:1/16:0(OH))**	
grey	Lipid	Secondary Bile Acid Metabolism	taurochenodeoxycholic acid 3-sulfate	HMDB0002486
grey	Lipid	Pregnenolone Steroids	pregnenetriol sulfate*	
grey	Lipid	Pregnenolone Steroids	pregnenetriol disulfate*	
grey	Lipid	Fatty Acid Metabolism (Acyl Glycine)	picolinoylglycine	HMDB0059766
grey	Peptide	Dipeptide	phenylalanylhydroxyproline*	HMDB0011176
grey	Peptide	Dipeptide Derivative	leucylhydroxyproline*	HMDB0028930
grey	Peptide	Dipeptide Derivative	isoleucylhydroxyproline*	HMDB0028908
grey	Xenobiotics	Food Component/Plant	vanillic acid glycine	HMDB0060026
grey	Amino Acid	Methionine, Cysteine, SAM and Taurine Metabolism	2-hydroxy-4-(methylthio)butanoic acid	HMDB0037115
grey	Amino Acid	Histidine Metabolism	1-methyl-5-imidazolelactate	
grey	Amino Acid	Methionine, Cysteine, SAM and Taurine Metabolism	S-carboxyethylcysteine	
grey	Partially Characterized Molecules	Partially Characterized Molecules	carnitine of C10H14O2 (5)*	
grey	Amino Acid	Lysine Metabolism	N-acetyl-2-aminoadipate	
grey	Lipid	Fatty Acid Metabolism (Acyl Glycine)	cis-3,4-methyleneheptanoylglycine	
grey	Partially Characterized Molecules	Partially Characterized Molecules	bilirubin degradation product, C16H18N2O5 (2)**	
grey	Partially Characterized Molecules	Partially Characterized Molecules	bilirubin degradation product, C17H18N2O4 (1)**	
grey	Partially Characterized Molecules	Partially Characterized Molecules	bilirubin degradation product, C17H18N2O4 (2)**	
grey	Partially Characterized Molecules	Partially Characterized Molecules	bilirubin degradation product, C17H18N2O4 (3)**	
grey	Partially Characterized Molecules	Partially Characterized Molecules	bilirubin degradation product, C17H20N2O5 (2)**	
grey	Partially Characterized Molecules	Partially Characterized Molecules	bilirubin degradation product, C16H18N2O5 (3)**	
grey	Amino Acid	Tryptophan Metabolism	oxindolylalanine	
grey	Xenobiotics	Chemical	perfluorohexanesulfonate (PFHxS)	HMDB0094700
grey			X-07765	
grey			X-11299	
grey			X-11308	
grey			X-11315	
grey			X-11372	
grey			X-11444	
grey			X-11470	
grey			X-11880	
grey			X-12007	
grey			X-12112	
grey			X-12193	
grey			X-12216	
grey			X-12410	
grey			X-12456	
grey			X-12462	
grey			X-12729	
grey			X-12812	
grey			X-12844	
grey			X-12906	
grey			X-13431	
grey			X-13684	
grey			X-13729	

grey			X-13866	
grey			X-15674	
grey			X-16935	
grey			X-16938	
grey			X-17010	
grey			X-17357	
grey			X-18779	
grey			X-18899	
grey			X-19141	
grey			X-21258	
grey			X-21286	
grey			X-21310	
grey			X-21339	
grey			X-21383	
grey			X-21410	
grey			X-21467	
grey			X-21471	
grey			X-21742	
grey			X-21796	
grey			X-21816	
grey			X-22771	
grey			X-23276	
grey			X-23636	
grey			X-23639	
grey			X-23644	
grey			X-23654	
grey			X-23739	
grey			X-24306	
grey			X-24307	
grey			X-24328	
grey			X-24541	
grey			X-24544	
grey			X-24549	
grey			X-24556	
grey			X-24576	
grey			X-24578	
grey			X-24951	
grey			X-24970	
grey			X-25172	
grey			X-25267	
grey			X-25343	
grey			X-25433	
grey			X-25519	
grey			X-25790	
grey			X-26054	
grey			X-26097	
grey			X-26119	
turquoise	Amino Acid	Tryptophan Metabolism	5-hydroxyindoleacetate	HMDB0000763
turquoise	Amino Acid	Tryptophan Metabolism	kynurenate	HMDB0000715
turquoise	Amino Acid	Tyrosine Metabolism	homovanillate (HVA)	HMDB0000118
turquoise	Amino Acid	Phenylalanine Metabolism	4-hydroxyphenylacetate	HMDB0000020
turquoise	Cofactors and Vitamins	Nicotinate and Nicotinamide Metabolism	quinolinate	HMDB0000232
turquoise	Amino Acid	Lysine Metabolism	N6,N6,N6-trimethyllysine	HMDB0001325
turquoise	Amino Acid	Polyamine Metabolism	N-acetylputrescine	HMDB0002064
turquoise	Amino Acid	Methionine, Cysteine, SAM and Taurine Metabolism	N-formylmethionine	HMDB0001015
turquoise	Amino Acid	Polyamine Metabolism	5-methylthioadenosine (MTA)	HMDB0001173
turquoise	Amino Acid	Phenylalanine Metabolism	2-hydroxyphenylacetate	HMDB0000669
turquoise	Amino Acid	Creatine Metabolism	creatinine	HMDB0000562
turquoise	Cofactors and Vitamins	Vitamin A Metabolism	retinol (vitamin A)	HMDB0000305
turquoise	Amino Acid	Urea cycle; Arginine and Proline Metabolism	urea	HMDB0000294
turquoise	Nucleotide	Pyrimidine Metabolism, Uracil containing	pseudouridine	HMDB0000767
turquoise	Nucleotide	Pyrimidine Metabolism, Cytidine containing	cytidine	HMDB0000089
turquoise	Nucleotide	Pyrimidine Metabolism, Orotate containing	dihydroorotate	HMDB03349
turquoise	Nucleotide	Purine Metabolism, (Hypo)Xanthine/Inosine containing	allantoin	HMDB0000462
turquoise	Cofactors and Vitamins	Pantothenate and CoA Metabolism	pantothenate	HMDB0000210
turquoise	Amino Acid	Methionine, Cysteine, SAM and Taurine Metabolism	N-acetylmethionine	HMDB0011745
turquoise	Amino Acid	Leucine, Isoleucine and Valine Metabolism	N-acetylvaline	HMDB0011757
turquoise	Amino Acid	Alanine and Aspartate Metabolism	N-acetylalanine	HMDB0000766
turquoise	Amino Acid	Tyrosine Metabolism	vanillylmandelate (VMA)	HMDB0000291
turquoise	Amino Acid	Polyamine Metabolism	4-acetamidobutanoate	HMDB0003681
turquoise	Nucleotide	Purine Metabolism, (Hypo)Xanthine/Inosine containing	urate	HMDB0000289
turquoise	Carbohydrate	Aminosugar Metabolism	N-acetylneuraminate	HMDB0000230
turquoise	Carbohydrate	Aminosugar Metabolism	N-acetylglucosaminylasparagine	HMDB0000489
turquoise	Amino Acid	Glutathione Metabolism	cys-gly, oxidized	
turquoise	Nucleotide	Purine Metabolism, Adenine containing	N1-methyladenosine	HMDB0003331
turquoise	Lipid	Phospholipid Metabolism	choline	HMDB0000097
turquoise	Peptide	Gamma-glutamyl Amino Acid	gamma-glutamylleucine	HMDB0011171
turquoise	Amino Acid	Histidine Metabolism	imidazole lactate	HMDB0002320
turquoise	Amino Acid	Tryptophan Metabolism	kynurenine	HMDB0000684
turquoise	Peptide	Gamma-glutamyl Amino Acid	gamma-glutamylphenylalanine	HMDB0000594
turquoise	Xenobiotics	Food Component/Plant	erythritol	HMDB0002994
turquoise	Amino Acid	Urea cycle; Arginine and Proline Metabolism	homocitrulline	HMDB0000679
turquoise	Amino Acid	Histidine Metabolism	1-methylhistidine	HMDB0000001
turquoise	Cofactors and Vitamins	Vitamin B6 Metabolism	pyridoxate	HMDB0000017
turquoise	Peptide	Gamma-glutamyl Amino Acid	gamma-glutamylvaline	HMDB0011172
turquoise	Amino Acid	Histidine Metabolism	1-methyl-4-imidazoleacetate	HMDB0002820
turquoise	Amino Acid	Phenylalanine Metabolism	N-acetylphenylalanine	HMDB0000512
turquoise	Amino Acid	Glycine, Serine and Threonine Metabolism	N-acetylthreonine	HMDB0062557
turquoise	Amino Acid	Leucine, Isoleucine and Valine Metabolism	N-acetylisoleucine	HMDB0061684
turquoise	Carbohydrate	Aminosugar Metabolism	erythronate*	HMDB0000613
turquoise	Nucleotide	Purine Metabolism, (Hypo)Xanthine/Inosine containing	N1-methylinosine	HMDB0002721
turquoise	Nucleotide	Purine Metabolism, Guanine containing	N2,N2-dimethylguanosine	HMDB0004824
turquoise	Nucleotide	Purine Metabolism, Adenine containing	N6-carbamoylthreonyladenosine	HMDB0041623
turquoise	Nucleotide	Pyrimidine Metabolism, Orotate containing	orotidine	HMDB0000788
turquoise	Nucleotide	Pyrimidine Metabolism, Uracil containing	5,6-dihydrouridine	HMDB0000497
turquoise	Nucleotide	Pyrimidine Metabolism, Uracil containing	3-(3-amino-3-carboxypropyl)uridine*	
turquoise	Nucleotide	Purine Metabolism, Guanine containing	7-methylguanine	HMDB0000897
turquoise	Cofactors and Vitamins	Nicotinate and Nicotinamide Metabolism	N1-methyl-2-pyridone-5-carboxamide	HMDB0004193
turquoise	Peptide	Gamma-glutamyl Amino Acid	gamma-glutamylisoleucine*	HMDB0011170
turquoise	Amino Acid	Leucine, Isoleucine and Valine Metabolism	2-methylbutyrylcarnitine (C5)	HMDB0000378
turquoise	Partially Characterized Molecules	Partially Characterized Molecules	glutamine_degradant*	
turquoise	Cofactors and Vitamins	Ascorbate and Aldarate Metabolism	gulonate*	HMDB0003290
turquoise	Amino Acid	Lysine Metabolism	glutarylcarnitine (C5-DC)	HMDB0013130
turquoise	Amino Acid	Leucine, Isoleucine and Valine Metabolism	tiglylcarnitine (C5:1-DC)	HMDB0002366
turquoise	Nucleotide	Purine Metabolism, Adenine containing	N6-succinyladenosine	HMDB0000912
turquoise	Amino Acid	Histidine Metabolism	1-ribosyl-imidazoleacetate*	HMDB0002331
turquoise	Lipid	Fatty Acid, Dihydroxy	3,4-dihydroxybutyrate	HMDB0000337
turquoise	Amino Acid	Lysine Metabolism	N6-acetyllysine	HMDB0000206
turquoise	Amino Acid	Glycine, Serine and Threonine Metabolism	N-acetylserine	HMDB0002931
turquoise	Energy	TCA Cycle	succinylcarnitine (C4-DC)	HMDB0061717
turquoise	Nucleotide	Pyrimidine Metabolism, Uracil containing	N-acetyl-beta-alanine	HMDB0061880
turquoise	Lipid	Fatty Acid, Dihydroxy	2R,3R-dihydroxybutyrate	HMDB0000498

turquoise	Amino Acid	Glutamate Metabolism	alpha-ketoglutaramate*	HMDB0001552
turquoise	Amino Acid	Glutathione Metabolism	cysteinylglycine disulfide*	HMDB0000709
turquoise	Cofactors and Vitamins	Ascorbate and Aldarate Metabolism	2-O-methylascorbic acid	HMDB0240294
turquoise	Xenobiotics	Food Component/Plant	mannonate*	
turquoise	Amino Acid	Methionine, Cysteine, SAM and Taurine Metabolism	lanthionine	
turquoise	Amino Acid	Histidine Metabolism	N-acetylcarnosine	HMDB0012881
turquoise	Amino Acid	Urea cycle; Arginine and Proline Metabolism	N2,N5-diacetylornithine	HMDB0240345
turquoise	Amino Acid	Methionine, Cysteine, SAM and Taurine Metabolism	methionine sulfone	HMDB0062174
turquoise	Xenobiotics	Chemical	O-sulfo-tyrosine	HMDB0155722
turquoise	Amino Acid	Methionine, Cysteine, SAM and Taurine Metabolism	N-acetyltaurine	HMDB0240253
turquoise	Amino Acid	Tyrosine Metabolism	tyramine O-sulfate	HMDB0006409
turquoise	Carbohydrate	Pentose Metabolism	arabonate/xylonate	HMDB0000539
turquoise	Amino Acid	Tyrosine Metabolism	vanillactate	HMDB0000913
turquoise	Amino Acid	Tryptophan Metabolism	C-glycosyltryptophan	HMDB0240296
turquoise	Carbohydrate	Pentose Metabolism	arabitol/xylitol	HMDB0001851,HMDB0000568,HMDB0002917
turquoise	Carbohydrate	Aminosugar Metabolism	N-acetylglucosamine/N-acetylgalactosamine	HMDB0000212,HMDB0000215
turquoise	Peptide	Acetylated Peptides	4-hydroxyphenylacetylglutamine	
turquoise	Amino Acid	Alanine and Aspartate Metabolism	hydroxyasparagine**	HMDB32332
turquoise	Amino Acid	Urea cycle; Arginine and Proline Metabolism	N,N,N-trimethyl-alanylproline betaine (TMAP)	HMDB0240365
turquoise	Amino Acid	Leucine, Isoleucine and Valine Metabolism	N-carbamoylvaline	
turquoise	Amino Acid	Urea cycle; Arginine and Proline Metabolism	3-amino-2-piperidone	HMDB0000323
turquoise	Cofactors and Vitamins	Ascorbate and Aldarate Metabolism	ascorbic acid 3-sulfate*	
turquoise	Amino Acid	Polyamine Metabolism	N-acetyl-isoputreanine	
turquoise	Amino Acid	Methionine, Cysteine, SAM and Taurine Metabolism	2,3-dihydroxy-5-methylthio-4-pentenoate (DMTPA)*	HMDB0240388
turquoise	Amino Acid	Lysine Metabolism	hydroxy-N6,N6,N6-trimethyllysine*	
turquoise	Peptide	Modified Peptides	N,N-dimethyl-pro-pro	
turquoise			X-11787	
turquoise			X-11979	
turquoise			X-12015	
turquoise			X-12026	
turquoise			X-12100	
turquoise			X-12104	
turquoise			X-12411	
turquoise			X-12713	
turquoise			X-12822	
turquoise			X-13553	
turquoise			X-15461	
turquoise			X-15503	
turquoise			X-17676	
turquoise			X-18887	
turquoise			X-22162	
turquoise			X-24337	
turquoise			X-24588	
turquoise			X-25371	
turquoise			X-25422	
turquoise			X-25810	
yellow	Lipid	Long Chain Polyunsaturated Fatty Acid (n3 and n6)	linoleate (18:2n6)	HMDB0006270,HMDB0000673
yellow	Lipid	Medium Chain Fatty Acid	laurate (12:0)	HMDB0000638
yellow	Lipid	Long Chain Polyunsaturated Fatty Acid (n3 and n6)	arachidonate (20:4n6)	HMDB0001043
yellow	Lipid	Long Chain Saturated Fatty Acid	palmitate (16:0)	HMDB0000220
yellow	Lipid	Long Chain Saturated Fatty Acid	stearate (18:0)	HMDB0000827
yellow	Lipid	Long Chain Monounsaturated Fatty Acid	palmitoleate (16:1n7)	HMDB0003229
yellow	Lipid	Long Chain Saturated Fatty Acid	myristate (14:0)	HMDB0000806
yellow	Lipid	Medium Chain Fatty Acid	caprate (10:0)	HMDB0000511
yellow	Lipid	Long Chain Saturated Fatty Acid	margarate (17:0)	HMDB0002259
yellow	Lipid	Long Chain Saturated Fatty Acid	nonadecanoate (19:0)	HMDB0000772
yellow	Lipid	Long Chain Saturated Fatty Acid	arachidate (20:0)	HMDB0002212
yellow	Lipid	Medium Chain Fatty Acid	caproate (6:0)	HMDB0000535
yellow	Lipid	Long Chain Saturated Fatty Acid	pentadecanoate (15:0)	HMDB0000826
yellow	Lipid	Long Chain Monounsaturated Fatty Acid	erucate (22:1n9)	HMDB0002068
yellow	Lipid	Long Chain Polyunsaturated Fatty Acid (n3 and n6)	dihomo-linoleate (20:2n6)	HMDB0005060
yellow	Lipid	Fatty Acid, Monohydroxy	2-hydroxystearate	HMDB0062549
yellow	Lipid	Glycerolipid Metabolism	glycerol	HMDB0000131
yellow	Lipid	Long Chain Polyunsaturated Fatty Acid (n3 and n6)	eicosapentaenoate (EPA; 20:5n3)	HMDB0001999
yellow	Lipid	Long Chain Polyunsaturated Fatty Acid (n3 and n6)	docosahexaenoate (DHA; 22:6n3)	HMDB0002183
yellow	Lipid	Fatty Acid, Monohydroxy	3-hydroxymyristate	HMDB0061656
yellow	Lipid	Monoacylglycerol	1-oleoylglycerol (18:1)	HMDB0011567
yellow	Lipid	Fatty Acid, Monohydroxy	3-hydroxydecanoate	HMDB0002203
yellow	Lipid	Monoacylglycerol	1-linoleoylglycerol (18:2)	HMDB0011568
yellow	Lipid	Fatty Acid, Monohydroxy	3-hydroxylaurate	HMDB0000387
yellow	Amino Acid	Leucine, Isoleucine and Valine Metabolism	3-hydroxy-2-ethylpropionate	HMDB0000396
yellow	Lipid	Long Chain Polyunsaturated Fatty Acid (n3 and n6)	docosapentaenoate (n3 DPA; 22:5n3)	HMDB0006528,HMDB0001976
yellow	Lipid	Long Chain Polyunsaturated Fatty Acid (n3 and n6)	docosadienoate (22:2n6)	HMDB0061714
yellow	Lipid	Long Chain Polyunsaturated Fatty Acid (n3 and n6)	adrenate (22:4n6)	HMDB0002226
yellow	Lipid	Long Chain Monounsaturated Fatty Acid	myristoleate (14:1n5)	HMDB0002000
yellow	Lipid	Long Chain Polyunsaturated Fatty Acid (n3 and n6)	stearidonate (18:4n3)	HMDB0006547
yellow	Lipid	Medium Chain Fatty Acid	5-dodecenoate (12:1n7)	HMDB0000529
yellow	Lipid	Long Chain Monounsaturated Fatty Acid	10-nonadecenoate (19:1n9)	HMDB0013622
yellow	Lipid	Long Chain Monounsaturated Fatty Acid	10-heptadecenoate (17:1n7)	HMDB0060038
yellow	Lipid	Long Chain Monounsaturated Fatty Acid	eicosenoate (20:1)	HMDB0002231,HMDB0062436
yellow	Lipid	Long Chain Polyunsaturated Fatty Acid (n3 and n6)	linolenate [alpha or gamma; (18:3n3 or 6)]	HMDB0003073,HMDB0001388
yellow	Lipid	Monoacylglycerol	1-arachidonylglycerol (20:4)	HMDB11578
yellow	Lipid	Fatty Acid, Monohydroxy	2-hydroxypalmitate	HMDB0031057
yellow	Lipid	Long Chain Polyunsaturated Fatty Acid (n3 and n6)	docosapentaenoate (n6 DPA; 22:5n6)	HMDB0001976
yellow	Lipid	Sterol	7alpha-hydroxy-3-oxo-4-cholestenoate (7-Hoca)	HMDB0012458
yellow	Lipid	Lysophospholipid	1-arachidonoyl-GPI (20:4)*	HMDB0061690
yellow	Lipid	Long Chain Polyunsaturated Fatty Acid (n3 and n6)	dihomo-linolenate (20:3n3 or n6)	HMDB0002925
yellow	Lipid	Lysophospholipid	1-linoleoyl-GPI (18:2)*	HMDB0240597
yellow	Lipid	Fatty Acid, Branched	(16 or 17)-methylstearate (a19:0 or i19:0)	HMDB0037397
yellow	Lipid	Medium Chain Fatty Acid	cis-4-decenoate (10:1n6)*	HMDB0004980
yellow	Lipid	Fatty Acid Metabolism (Acyl Glycine)	N-palmitoylglycine	HMDB0013034
yellow	Lipid	Lysoplasmalogen	1-(1-enyl-oleoyl)-GPE (P-18:1)*	
yellow	Lipid	Fatty Acid, Monohydroxy	9-hydroxystearate	HMDB0061661
yellow	Lipid	Sterol	3beta-hydroxy-5-cholestenoate	HMDB0012453
yellow	Lipid	Endocannabinoid	linoleoyl ethanolamide	HMDB0012252
yellow	Lipid	Long Chain Monounsaturated Fatty Acid	oleate/vaccenate (18:1)	HMDB0003231,HMDB0000573,HMDB0240219,HMDB0000207
yellow	Lipid	Long Chain Polyunsaturated Fatty Acid (n3 and n6)	hexadecadienoate (16:2n6)	HMDB0000477
yellow	Lipid	Fatty Acid, Monohydroxy	2-hydroxybehenate	HMDB0061660
yellow	Lipid	Long Chain Polyunsaturated Fatty Acid (n3 and n6)	tetradecadienoate (14:2)*	HMDB0000560
yellow	Lipid	Fatty Acid, Dicarboxylate	dodecadienoate (12:2)*	
yellow	Lipid	Medium Chain Fatty Acid	(2 or 3)-decenoate (10:1n7 or n8)	
yellow	Partially Characterized Molecules	Partially Characterized Molecules	branched-chain, straight-chain, or cyclopropyl 10:1 fatty acid (1)*	
yellow	Partially Characterized Molecules	Partially Characterized Molecules	branched-chain, straight-chain, or cyclopropyl 12:1 fatty acid*	
yellow			X-21353	
yellow			X-23782	
